# Large Doses of Sulphate of Quinine, in Malignant Intermittents

**Published:** 1837

**Authors:** 


### Art. II.—Large Doses of Sulphate of Quinine in Malignant Intermittents.

In the Transylvania Journal for July, 1837, we find an Inaugural dissertation, by Dr. J. E. May, of North Alabama, on the beneficial influence of large doses of sulphate of quinine in certain forms of intermittent. The author, guided by the experience of the very respectable Dr. Fearn, and other practitioners along the Tennessee river, where malignant intermittents are endemic, has given the sulphate in fifteen grain doses, at short intervals, in the decline of the paroxysm, with the happiest effects. Sometimes he has administered three or four scruples in a single intermission. Occasionally, he, and the gentlemen on whose greater experience he relies, have united calomel with the quinine, and found the combination decidedly beneficial.

We are pleased to meet with this new testimony to the safety and value of large doses of the sulphate. Dr. M. seems to regard them as without precedent; but in this he is mistaken. At least ten years ago, Dr. Perrine, of the State of Mississippi, administered this medicine in such quantities as to amount to a drachm, in a single intermission; and we, ourselves, for many years past, have been accustomed to give it in doses of ten or fifteen grains. We have, also, been in the habit of combining it with calomel, a practice which is general among the physicians of this quarter. Of the harmlessness of large doses, we some time since had conclusive evidence, by being called to see a man, the next day after he had, by mistake, taken a drachm at one portion. He still had a roaring in his ears, but was walking about the house, and had not experienced any formidable symptoms. They who limit themselves to small doses at short intervals, are not aware how much, *in many cases*, they sacrifice to their timidity. To a patient who labored under a neuralgic affection, apparently of miasmatic origin, we lately, in conjunction with a medical friend, administered a scruple daily, for five or six weeks, without any other sinister effect, than a noise in the head and a slight degree of deafness. It was given in five grain doses, combined with ten grains of nitrate of potash. D.

